# Utilizing health information technology in the treatment and management of patients during the COVID-19 pandemic: lessons from international case study sites

**DOI:** 10.1093/jamia/ocab057

**Published:** 2021-03-13

**Authors:** Stephen Malden, Catherine Heeney, David W Bates, Aziz Sheikh

**Affiliations:** 1 Centre for Medical Informatics, Usher Institute, University of Edinburgh, Edinburgh, UK; 2 Division of General Internal Medicine, Brigham and Women’s Hospital, Boston, MA, USA; 3 Department of Health Policy and Management, Harvard Chan School of Public Health, Boston, MA, USA

**Keywords:** Electronic health records, Medical informatics, Patient safety, Quality of health care, Telemedicine

## Abstract

**Objective:**

To develop an in-depth understanding of how hospitals with a long history of health information technology (HIT) use have responded to the COVID-19 pandemic from a HIT perspective.

**Materials and methods:**

We undertook interviews with 44 healthcare professionals with a background in informatics from six hospitals internationally. Interviews were informed by a topic guide and were conducted via videoconferencing software. Thematic analysis was employed to develop a coding framework and identify emerging themes.

**Results:**

Three themes and six sub-themes were identified. HITs were employed to manage time and resources during a surge in patient numbers through fast-tracked governance procedures, and the creation of real-time bed capacity tracking within electronic health records. Improving the integration of different hospital systems was identified as important across sites. The use of hard-stop alerts and order sets were perceived as being effective at helping to respond to potential medication shortages and selecting available drug treatments. Utilizing information from multiple data sources to develop alerts facilitated treatment. Finally, the upscaling/optimization of telehealth and remote working capabilities was used to reduce the risk of nosocomial infection within hospitals.

**Discussion:**

A number of the HIT-related changes implemented at these sites were perceived to have facilitated more effective patient treatment and management of resources. Informaticians generally felt more valued by hospital management as a result.

**Conclusions:**

Improving integration between data systems, utilizing specialized alerts, and expanding telehealth represent strategies that hospitals should consider when using HIT for delivering hospital care in the context of the COVID-19 pandemic.

https://mc.manuscriptcentral.com/jamia

## BACKGROUND

The novel coronavirus disease 2019 (COVID-19), which causes severe respiratory disease in high-risk groups[1], was first identified in Wuhan in China and has subsequently progressed to a global pandemic causing over 100 million infections and almost 2.3 million deaths as of February 2021[2]. Despite unprecedented measures to control the spread of the virus[3], prevalence remains high, and health services have been under considerable strain as a result[4-7]. For example, one meta-analysis found that 32% of patients entering hospital due to COVID-19 were subsequently admitted to intensive care units (ICU)[8]. Additionally, hospitals have experienced significant increased strain due to drug shortages[5], ICU resource shortages[9], and infection of frontline healthcare personnel[4].

While long-term solutions to the pandemic will likely be based on the roll-out of vaccines and effective treatments [10, 11], a number of researchers and practitioners have highlighted the role that electronic health records (EHRs), computerized physician order entry systems (CPOE), and telehealth can play in managing and treating the high volume of hospital admissions and cases effectively [12-16], which is typically managed by medical informaticians. Such technologies have already been successfully utilized to manage patients during the ongoing COVID-19 pandemic. For example, reports and studies have advocated for the role telehealth could play in reducing risk of both provider and routine patient exposure to the virus [17-21]. Additionally, using data from EHRs to identify potential cases and manage workflow has been highlighted as a potential strategy, as has the customization of provider screens, clinical decision support (CDS) and efforts to increase interoperability of different systems within the wider EHR [13, 14, 22].

## OBJECTIVES

Health systems in economically-developed countries have seen increased uptake of EHR implementation and related health information technology (HIT) in recent years[23, 24]. However, https://mc.manuscriptcentral.com/jamia identifying approaches to appropriately and effectively use such technology to combat the burden of COVID-19 requires knowledge sharing. Therefore, the aim of this qualitative study was to identify HIT strategies used by digitally mature hospitals in direct response to COVID-19 and make recommendations based on these approaches.

## METHODS

This paper forms part of a larger ongoing qualitative study investigating the best approaches to optimizing CPOE based on the insights of key personnel within digitally mature hospitals in the United Kingdom (U.K.), mainland Europe and the United States (U.S.). The methods employed are described in detail elsewhere[25], but the methodology is described briefly below.

### Recruitment

Participants were healthcare professionals or informatics specialists drawn from digitally mature sites (defined using the latest HIMSS Electronic Medical Record Adoption Model criteria as hospitals classified as level six or seven)[26] that had been extensively involved in the implementation and optimization of HITs such as EHRs, CPOE, CDS and telehealth. Sites were identified through two distinct strategies: firstly, a scoping review of optimization strategies in electronic prescribing was undertaken[27], with sites featuring prominently in this literature contacted for participation. Additionally, drawing on a network of experts in the field of electronic prescribing, potential sites were discussed as candidates for recruitment at roundtable events. Following the identification of six digitally mature sites, emails were sent to key contacts (such as pharmacy managers, or heads of research departments) to request permission to contact members of staff within the organisation. Following this, a purposive sampling strategy was used to recruit key member of staff who had been involved in optimizing HIT within each site, with information and consent forms communicated via email.

https://mc.manuscriptcentral.com/jamia

**Data collection**

Semi-structured interviews were used in this study, with interviews conducted by two experienced qualitative researchers (CH and SM). The interviews primarily focused on general approaches to optimizing CPOE. However, as the COVID-19 pandemic arose immediately prior to commencement of data collection, questions regarding each site's response to the pandemic from a HIT standpoint were incorporated into the broader interview topic guide. Specifically, participants were asked whether any specific changes or strategies were put in place within the EHR or related technologies as a direct response to COVID-19. When asking questions relating to telehealth, this was defined as any form of healthcare that was provided remotely to a patient. Follow-up questions were then asked based on the participant's responses to gain further insights. All interviews were conducted via videoconferencing software. All interviews were audio-recorded and transcribed verbatim. The full interview topic guide can be viewed in supplementary file 1, with questions 5, 5.1 and 5.2 relating to this study.

**Data analysis**

Thematic analysis was undertaken on the dataset using an inductive approach[28], which involved the coding of transcripts to identify themes. In order to develop a coding framework, two researchers (CH and SM) first independently coded two transcripts before discussing any discrepancies between code allocations in the text. The framework was then applied to the remaining transcripts, before the researchers collectively grouped codes into themes and sub-themes. In order to reduce the potential effects of researcher biases during data collection and analysis, the researchers employed prospective reflexivity when conducting the interviews and interpreting the findings[29]. Data analysis was conducted using NVivo 12 pro qualitative data analysis software. Further details on data collection and analysis are available elsewhere[25].

**Institutional review board approval**

https://mc.manuscriptcentral.com/jamia

Ethical approval for this study was granted by the Usher Research Ethics Group (University of Edinburgh) on 21/01/2020 (ref.1906). Further details of ethical considerations of the study are detailed elsewhere[25].

**RESULTS**

Forty-four interviews were conducted across six sites (two U.K. and four U.S.-based sites) all of which were teaching hospitals affiliated with academic institutions. A range of healthcare professionals, all with expertise in medical informatics, participated in the interviews. Interviewee roles included pharmacy managers, pharmacists, nurses, physicians, data analysts and chief information officers. Further details of each site are presented in Table 1.

Table 1. Site and interview participant details.

| | Hospital details | | | Participant details | | EHR system details | | |
|---|---|---|---|---|---|---|---|---|
| Site identifier | Location | Size | Type | Roles included in sample | Total number | Vendor or home-grown | Integrated or best of breed (BoB) | COVID-19 related optimizations identified |
| Site 1 | UK | ~760 beds | Teaching hospital | Pharmacy managers, analysts, pharmacists, nurses, information officers | 6 | Vendor | BoB | Combining data sources, CDS, alerts, telehealth, remote ward rounds |
| Site 2 | UK | ~800 beds | Teaching hospital | Pharmacy managers, physicians, analysts, pharmacist, Nurses, | 13 | Vendor | Integrated | Expediting the transition of paper-based processes to control contagion and to allow |

| | | | | | | | | |
|---|---|---|---|---|---|---|---|---|
| | | | | Other Ancillary care | | | | remote working |
| Site 3 | US | ~800 beds | Teaching hospital | Pharmacy managers, physicians, analysts, pharmacists | 8 | Vendor | Integrated | Integration and interoperability of systems, CDS, modified alerts, telehealth |
| Site 4 | US | ~670 beds | Teaching hospital | Physicians, nurses | 3 | Home-grown | BoB | CDS, EHR assisted infection control and discharge |
| Site 5 | US | ~1500 beds | Teaching hospital | Pharmacy managers, physicians, pharmacists Information officers | 5 | Vendor | Integrated | CDS, integration and interoperability of systems, modified alerts, remote working, telehealth |
| Site 6 | US | ~80 beds | Paediatric Cancer hospital | Pharmacy managers, physicians, analysts, information officers | 9 | Vendor | Integrated | Alerts to inform physicians of COVID/testing status of patients. Adding PCR tests /drugs to the system |

Thematic analysis identified three major themes and six sub-themes, detailed below. Further quotes can be observed in table 2.

**Managing time and resources**

One major theme discussed by all participants was the need to manage an unusually high number of patients, coupled with the additional strain the pandemic placed on resources such as medications, ICU beds, and staff. This necessitated rapid and responsive organizational and technological changes to health systems.

*Expediting governance processes*

https://mc.manuscriptcentral.com/jamia

The pandemic was viewed across study sites as impacting on the ordinary governance procedures involved when seeking to change practices or alter HIT systems rapidly. This was viewed as largely positive by those seeking to move existing paper-based practices to digital, especially given the time and safety considerations raised by the pandemic. Putting streamlined governance in place facilitated timely implementations and optimizations of new HIT adaptations related specifically to managing COVID-19.

> *We introduced a system whereby anything COVID related moved up the list as it were, and there has been, sort of the [site] set up the different sort of communications network to deal with COVID. So, requests that come in around COVID could be dealt with, you know, quicker than they would have done normally, so all that's been done.*

Site 2. Informatics lead (U.K.)

In the following excerpt, the interviewee is describing changes to the system made in response to a government imperative to decrease the burden of documentation prompted by COVID-19.

> *So, what we did was we took a look at the regulations and said, okay, well we can make some changes within our system. Some of it was just that we told people that they didn't have to get the patient to sign it any more. But then we were able to, you know, make some simple changes within our system to designate areas where nurses did need to document.*

Site 4. Informatics Nurse (U.S.)

It was apparent across study sites that in addition to streamlining governance procedures, informaticians were also given more autonomy and leveraged to develop HIT solutions to the issues created by COVID-19. For example, hospital management were perceived to have taken more interest in the potential of EHRs and the use of data to facilitate more efficient and targeted care and make changes rapidly. Some participants stated that they felt their work was more valued by management than before the pandemic.

https://mc.manuscriptcentral.com/jamia

*With the current situation, we have had some of the attention of those other people [hospital management] because it's become more of a management issue, which patient is going in which ward is affected by their COVID status, that sort of thing. And preventing, you know, people moving around the hospital, getting data to do it automatically is more of a focus for hospital management than it has been in the past. So, you know, that's a sort of positive thing that's come out of it in a way.*

Site A. Infromatician (U.K.)

*Longer-term impact*

A number of interviewees that acknowledged the rapid shifts both to the CPOE system and in the behavior of users, also questioned what the longer-term impact of the pandemic would be.

*But, you know, the comprehensiveness of the system is, you know, basically all order comms, patient vital sign recording, yes, nursing documentation, medical documentation. All that is on the system now. And even, you know, COVID has accelerated some change. I wonder how we will pull back or decide what to keep when it's all over.*

Site 2. Pharmacy manager (U.K.)

*Managing hospital beds*

Participants highlighted that general and critical care beds were near capacity during the first wave of the pandemic, and measures were needed to control flow of patients between admission, ICU, and discharge. One such example was the incorporation of discharge assessment into the EHR system, which allowed discharging physicians to complete the necessary patient checklists that would ordinarily be completed separately by infection control; this significantly reduced discharge times and, in turn, increased hospital bed capacity.

> *To take someone off precautions, to be able to move them out of their room or out of the hospital, we had to have infection control review their record. So I would have to pick up the phone and say…and have a conversation, so even just that, we were able to build a de-escalation order that had a series of yes, no questions that we basically modelled on what the infection control person would have been asking… de-escalation orders usually took 12 hours to get done to like two hours, which has a big impact on patient throughput…*
>
> Site 4. Physician A (U.S.)

Additionally, EHRs and related functionalities were deemed to have been vital in managing the high number of patients and forecasting which wards would soon be reaching capacity. Improving interoperability of different systems was an important step to achieving this.

> *Identifying the appropriate infection control precautions for a patient at the time of admission and then linking that to reports around bed utilization so that we can much easier see how many patients are in COVID precautions in the hospital, how many patients are positive. And you can have really a window in terms of your bed efficiency and blocked beds, if you need private rooms for COVID positive patients. And so that really drove a large system change around linking bed assignments, electronic infrastructure for bed assignments, to orders for precautions, to lab results.*
>
> Site 4. Physician B (U.S.)

*Reducing medication shortages*

Most participants acknowledged that the first wave of the pandemic placed considerable strain on medication supplies, with providers often ordering drugs that had not yet been shown to be https://mc.manuscriptcentral.com/jamia effective in treating COVID-19 patients, in turn leading to shortages of treatments for other conditions. Once inappropriate prescribing patterns were identified, two of the U.S. sites developed hard-stop alerts, which prevented patients confirmed to be COVID-19 positive from being prescribed certain drugs through the CPOE system, for example tocilizumab.

> *The best example I can tell you is tocilizumab, an IL-6 inhibitor used all the time for rheumatoid disease and sometimes used for other oncologic indications for cytokine storm not related to COVID. But people were using it for COVID and we were starting to have drug shortages and all kinds of other things. It was like, we've got to block this from COVID ordering. So, if the patient was COVID positive and ordered tocilizumab it blocked them but if they ordered tocilizumab for a patient who was not COVID positive, [the system] allowed it to go through.*

Site 3. Pharmacy Manager (U.S.)

In the case that inappropriate ordering was undertaken, some sites utilized order sets in combination with decision support to both advise on correct prescribing, but also to point to alternatives to the drugs prescribed if supply was limited.

> *We used order sets to at least flag labs and imaging quickly because everyone is learning all of it, and so we used admission order sets and order sets a little bit more to drive people to at least think about things. We were able to use decision support programming to help with Remdesivir when it was still limited, to drive indications and help identify the appropriate patients for its use.*

Site 4. Physician B (U.S.)

**Improving treatment accuracy and effectiveness**

*Interoperability of systems and integration of data sources*

https://mc.manuscriptcentral.com/jamia

Making all relevant information about a patient easily accessible and viewable to providers was highlighted as important when treating a high number of patients with COVID-19 and other complex morbidities. Examples of configuring provider computer screens to allow this information to be viewed easily were provided in two sites, and the capabilities of integrated EHR systems was exploited to allow for the combining of data from multiple sources such as labs and prescribing.

> *A snapshot screen that they've created for COVID where basically all of the relevant laboratory parameters and clinical information you'd want to know about a COVID patient, are all brought together in one screen which feature in [integrated EHR system] a lot of specialities. You know, when it was needed in the in-patient setting, I think, rather quickly and it was mentioned by many clinicians to be very useful in the care of complex COVID patients.*

Site 3. Physician (U.S.)

Similarly, building patient information from multiple systems into CDS messages in order to identify patients who may require a higher level of care was also undertaken within numerous sites. This was viewed as a way of facilitating timely, targeted treatment for patients who may need further surveillance for complications.

> *With acute kidney injury, when patients come in, we pull data from the pathology labs, we pull data from prescribing, and we can flag to our pharmacists, actually, this patient's at really high risk of acute kidney injury, and we're doing that with COVID patients now, that are coming in, 'cause we know that they are susceptible to renal failure, so you know, we can start to flag those patients nice and early.*

Site 1. Pharmacy Manager (U.K.)

https://mc.manuscriptcentral.com/jamia

**Negating infection risk in hospital**

Across all study sites, EHRs and related technologies played a major role in identifying COVID-19 positive patients, coordinating isolation units, and minimising infection risk to both providers and patients being treated for other conditions. For example, after receiving a positive test result, or displaying COVID-related symptoms, changes were made to provider display screens to clearly indicate when a patient had been confirmed or was suspected to have COVID-19.

> *A big thing that we were involved with was how to alert our providers that a patient is COVID-19 positive. We…have a blue banner that changes. So, it's like the header, top of the screen…it has a lot of patient information. Height, weight, allergies information. It turns blue if a patient is suspected COVID or COVID positive.*

Site 5. Pharmacy manager (U.S.)

The need to ensure patients were being placed in the correct part of the hospital and to keep track of patients within the hospital has proven to be an important functionality of the EHR system.

> *Yeah, there's been plenty of impacts because of COVID. I think the main thing has been more about identification, streaming of patients to the correct zones in the hospital, being able to quickly identify and locate where said patients are, whether their status is, in fact, probably or confirmed.*

Site 2. Physician (U.K.)

*Upscaling of telehealth usage and capabilities*

Moving routine and non-essential outpatient appointments to telehealth (either via phone call, video-conferencing, or existing patient portals) was undertaken to some degree within all six of the https://mc.manuscriptcentral.com/jamia participating sites, and this was highlighted as an integral aspect of reducing risk of infection to both patients and providers.

> *Like tele-medicine, through the patient portal, before COVID we were doing about 30 tele-medicine visits a week, and now we're over 4,000 a day. Our adoption…of a patient portal, it went from 30 per cent to about 75 percent.  COVID has, you know, completely changed things.*

Site 5. CMIO (U.S.)

Similarly, using technology to facilitate minimal-contact ward rounds within hospital was implemented in multiple sites, which reduced the need to use personal protective equipment (PPE), and mitigated risk of infection to providers.

> *We put monitors in rooms so that I could actually contact the patient, see the patient from outside their room so we didn't have to use so much PPE going in and out of rooms so that way, kind of consolidating how many times I went into a patient's room.  So we had those set up in our intensive care units and in our…in what we designated as our infection or COVID units.*

Site 5. Informatics manager (U.S.)

Such capabilities were also extended to allow many routine procedures such as medication reconciliation and patient chart updates to be conducted remotely, often outside the hospital campuses via secure remote-working systems. This was viewed as an important aspect of risk de-escalation for providers who would otherwise need to enter wards to conduct such tasks. Additionally, staff who were required to self-isolate due to a positive close-contact, could still provide support remotely by conducting medication reconciliation or administration from home.

> *During this pandemic, it's been really useful, because we've been able to, even when staff are self-isolating, they can still cover a ward remotely, 'cause we can remote into the summary*

https://mc.manuscriptcentral.com/jamia

*care record, we can remote into their prescribing record, and all the other systems, so we've been covering lots of the hospital, doing all the medicines reconciliation, all that kind of stuff remotely from home.*

Site 1. Pharmacy manager (U.K.)

Table 2. Additional quotes for the themes and sub-themes identified in the study

| **Theme/**sub-theme | **Quotes** |
|---|---|
| **Managing time and resources** | |
| Expediting governance processes | *for years we've been trying to do the [Take-home medications] process electronically and it's met with resistance from doctors and pharmacists. You know, they're like, oh, it'll never work, sort of scenario. And so we had something almost ready to go that would fit and then with the COVID it went in a week because everyone could see that sort of thing needed to be done.* Site1. Informatics nurse (U.K.)<br><br>*it took only six...it took a matter of hours to implement something, a change to the system, when it was very urgent related to patient care, but if it's something that's not as urgent it could take much longer.* Site 3. Pharmacist (U.S.)<br><br>*now that we've had COVID it works even better [governance process] because now we've had to make so many changes so quickly. We had that structure originally and people were familiar with each other already and now they trust each other a lot more.* Site 5. Pharmacy manager (U.S.)<br><br>*So, we have some bright people who were given the charge to do something very quickly, they are very bright people and, in fact, it did get done very quickly. As soon as they got that done, another request came along to do something else, you know, and can you do that by next Wednesday, kind of a thing.* Site 6.CDSmanager (U.S.) |
| Longer term impact | *I think it's going to be really interesting to see what the new normal looks like, when the service is back up and running. Clearly, it's really important for us to speak to patients. We want to speak to patients. But, yeah, there's kind of no reason why people can't be doing that in different ways, and actually if we can get the Microsoft Teams bit working at the patient bedside, so actually you could speak to patients as well, and then in many ways we could start to deliver our service in a hugely different way.* Site 1. Pharmacist (U.K.) |
| Managing hospital beds | *for the discharge medication for a patient to go…whatever they're going home on, it used to be prescribed on probably two Maxims on [EHR system] and then printed out by the doctor and then sent down on a piece of paper down to pharmacy and they would dispense from that piece of paper. So now it's still done on [EHR system], a button is hit to say the patient's ready to go home and it's printed off. It is still printed off in pharmacy but at least it's there, rather than having to go through the process. So that was…I mean, that's quite a major change.* Site 1. Informatics nurse (U.K.) |

| | |
|---|---|
| | *there's been plenty of impacts because of COVID. I think the main thing has been more about identification, streaming of patients to the correct zones in the hospital, being able to quickly identify and locate where said patients are, whether [they are], in fact, you know, probably or confirmed [COVID-19].* Site 2. Physician (U.K.) |
| Reducing medication shortages | *what I've seen that's been helpful to providers are these best practice alerts that come up that inform providers on things that come up like say for drug shortages or for recommendations for the COVID that are, you know, kind of, acute things that help providers guide them into the right thing saying, oh, we don't have this medication, we're switching over to this, so that these types of alerts that keep people, kind of, up to date on what's been recommended have been, I think, helpful on that.* Site 3. Pharmacist (U.S.)<br><br>*Tocilizumab…so when this information was coming out quickly one thing they did do is they put restrictions on who could order it to prevent, you know, rogue prescribing in the absence of data. So that was like one of the things that they did as far as a quick thing that they could make a change in the background and limit who was getting it.* Site 3. Physician. (U.S.)<br><br>*Most of our alerts were around drug shortages and the different mitigation strategies on, and then which drugs we were using because we were experiencing drug shortages so quickly that it was almost every day we were building alerts or firing something different to guide providers.* Site 5. Pharmacy manager (U.S.) |
| **Improving treatment accuracy and effectiveness** | *if someone tries to prescribe Hydroxychloroquine, which has now shown that it's not very evidenced base to use, they'll get an alert that says, oh, we don't use that here for COVID. We just...it's, you know, used only for rheumatologic or...so they've tried to optimise in terms of some recommendations.* Site 3. Pharmacist (U.S.)<br><br>*So we were able to identify drugs that needed to be approved or not approved if the patient was on COVID. So if I had a patient who was COVID positive, that BPA [best practice alert] would fire when they would try to order anything nebulisation, Albuterol, inhaled Epoprostenol, and said, you will not be able to order this, please use the metered dose inhaler. So we built a lot of decision support based on COVID-19 results, whether they're anticoagulation, treatment algorithms, I mean, really, they were…we did a lot, we worked daily on COVID updates.* Site 5. Pharmacy manager. (U.S.) |
| Interoperability of systems and integration of data sources | *I'm working on, since the COVID situation, an oxygen report to try and look at oxygen usage from a moment to moment basis, and we're using a bit of [CPOE], bit of our critical care [CPOE] system, a bit of our [CPOE] system to be able to pull that data together. We wouldn't have been able to do that not very long ago. Each new database that we get access to allows…is another piece of the puzzle really, that allows us to bring those things together.* Site 1. Analyst (U.K.)<br><br>*we were able to optimise this report and get it automatically run and emailed to our entire pharmacist staff every 24 hours. So this is a way for the frontline pharmacists that are working in the COVID ICUs, even some of the med surg units where we have, you know, some COVID patients, they can see, hey this patient is blowing through Midazolam, to help us manage our supply. Are* |

| | |
|---|---|
| | *there more clinical alerts we need to put? Do we need to put in any hard stops for Midazolam?* Site 3. Pharmacy manager (U.S.) |
| **Negating infection risk in hospital** | *They [neighboring hospital] haven't got [CPOE]. So their level of service, has been so much worse, and yet their pharmacy team has been more effected by COVID as in, you know, their team contracting it, because they're having to go to wards, whereas we've been able to not go to wards, but still offer a much more robust service.* Site 1. Pharmacist (U.K.)<br><br>*some patients don't have their own devices or they're so sick they can't manage their own device and so having the iPads deployed was phenomenal and we use that. Physicians used it to talk with the families and then at end of life it was also a very good resource. Site 5. Informatician (U.S)*<br><br>*Some things at the beginning when we were trying to socially distance, when people were working from home and they're self-isolating having an electronic system has been brilliant because they can work from home. They're socially isolating, they're not unwell, so they can do a clinical job from home. We had people sort of paired up so have one person at home and one person on the floor so if they needed anything on site they were liaising. But that has really helped; it's helped with doctors for doing remote clinics, we've still been able to get prescriptions generated.* Site 2. Pharmacy lead (U.K.)<br><br>*I know there are a lot of different alerts that are firing that inform physicians, they inform practice nurses of patients, for example, is either COVID positive or has not been tested and they have to be tested or they are in quarantine until the result comes back.* Site 6. Pharmacist (U.S.) |
| Upscaling of telehealth usage and capabilities | *we've got a lot of people working from home, so when we were looking at our approach there we quite deliberately, rather than bringing in a new home working remote access solution, we took the solutions that we'd got and scaled them out. One of the benefits of that was…one of the solutions is a virtual display approach, which we had because our particular clinical system only worked that way. What that's meant is that clinicians can actually also work at home.* Site 1. CMIO (U.K.)<br><br>*so before COVID, yes, getting people to use the [patient] portal, oh, was like painful but we have over 70 per cent and I'm sure it's even higher now, people that are now using the portal so we have seen a dramatic change with COVID.* Site 5. Informatician (U.S.) |

## DISCUSSION

We assessed HIT-dependent approaches used to treat patients, manage resources, and mitigate in-hospital infection risk during the COVID-19 pandemic within six digitally mature international sites. All participating sites acted quickly in altering governance and normal procedural practices to allow rapid changes to be made to EHR systems and accompanying technologies. Changes to such organizational factors have been highlighted in the literature as integral to responding to the pandemic[14]. Additionally, making changes to increase interoperability of systems, ease of access to and presentation of relevant patient data, and upscaling of telehealth usage were all adaptations viewed as facilitating more effective practice in the present study. Importantly, the HIT-related responses to COVID-19 were perceived to have further highlighted the value of informaticians for improving care and responding quickly to emergent needs.

The optimization and addition of new alerts were utilized both to improve treatment effectiveness, and to reduce drug shortages experienced due to incorrect prescribing by introducing "hard stop" alerts in combination with decision support and order sets, directing prescribers to more appropriate medications. Similar strategies have been employed to decrease drug shortages prior to the COVID-19 outbreak. For example, Brokenshire and colleagues tested a combination of hard and soft-stop alerts to address opioid shortages[30]. As expected, the study found hard stops to be more effective at reducing opioid ordering with immediate effect, while soft stops led to more subtle decreases in medication ordering over time or had no effect. While the use of hard stop alerts must be carefully considered in routine medical care[31], their ability to abruptly stop inappropriate ordering of scarce medications may be of most benefit during pandemic situations where the strain on resources is unprecedented, as demonstrated by their adoption in this present study.

Another healthcare resource which participating sites used HIT to manage were hospital beds, both through the building of discharge procedures into the EHR and ensuring appropriate integration of systems and data sources to allow bed capacity to be managed in real-time. Both these strategies have also been described in the literature regarding responses to COVID-19[32]. Additionally, other https://mc.manuscriptcentral.com/jamia innovative technological approaches to ensuring patient flow and bed availability are evident in the literature. One study aimed to decrease the burden of occupied beds by fitting wearable devices to 40 patients at discharge to monitor vital signs[21]. While many patients required home oxygen orders, none were re-hospitalised in this study. Such initiatives, coupled with streamlined EHR-based discharge assessment and increased system integration, have helped make available badly needed hospital beds.

In addition, HIT was not only used to treat COVID-19 patients and manage the direct burden on resources, but also to mitigate the risks of infections occurring within hospital. Telehealth was utilized to some extent in all sites, with hospitals that already had some form of patient portal upscaling the use of such technologies. In many places, this was dramatic, with near overnight adoption of telehealth at a scale not previously considered. The potential for telehealth to protect both routine patients and healthcare personnel from infection is highlighted by Russi and colleagues[33]. Additionally, the rapid implementation of a patient self-triage tool within a EHR patient portal was effective at reducing non-urgent triage times, in-person contacts, and had 88% sensitivity for detecting emergency-level patients[34].

The present study resonates with the literature in highlighting the use of mobile devices and screens to limit face-to-face contact between healthcare staff and confirmed COVID-19 patients as part of telehealth provision. The use of bedside tablets in isolation units, and remote medication reconciliation procedures in the present study were both considered important interventions in reducing risk of infection amongst participants. Similarly, Lin et al. (2020) utilized tablets at patient's bedsides for physical examinations, remote familial visits, and non-COVID-19 related care such as new born-family interactions, and remote group therapy sessions[14]. Grange et al. (2020) also describe the use of a cart-mounted camera and screen used to monitor ICU patients with COVID-19, which allowed specialists to see patients remotely, and removed the need to don PPE which is both time-consuming and costly[22]. However, such technologies must be adequately integrated with the https://mc.manuscriptcentral.com/jamia relevant existing systems and patient data sources if an optimized outcome for care is to be ensured. Furthermore, isolating patients can both be difficult psychologically and may also increase their safety risks[35].

This study has several limitations that should be considered when interpreting the findings. Firstly, we present early qualitative findings without accompanying outcome evaluations of the technological changes described by the participants in this study. It is therefore not possible to draw definite conclusions regarding the effectiveness of these interventions, and formal evaluations should be undertaken to determine efficacy. Secondly, this paper presents findings obtained from interviews that were primarily designed to investigate the wider optimizations of CPOE systems at the participating sites[25] rather than specific optimizations due to COVID-19, meaning the data obtained may not fully capture the full extent of the adaptations undertaken. Despite this, the present study included perspectives from over 40 experts in medical informatics and patient safety within digitally mature sites, who were all directly involved in the technological adaptations described to some extent. Furthermore, while similar studies have previously been published investigating the use of HIT to respond to COVID-19[15, 17, 18, 21, 22, 32, 34], these studies are largely small-scale, single centre studies. In contrast, this study presents data from six digitally mature sites both in the U.S. and U.K; offering further lessons of the role informatics can play in combating COVID-19 across differing healthcare contexts. The present study also further highlights the value of informaticians, not only in how they can contribute to optimal routine patient care, but also how they can rapidly configure and implement relevant HITs in acute situations such as the COVID-19 pandemic.

## CONCLUSIONS

This study presents the views and experiences of key experts in patient safety and medical informatics who have worked to improve their site’s respective EHR systems during the COVID-19

https://mc.manuscriptcentral.com/jamia pandemic. As might be expected, many systems made substantial and rapid changes to adapt which were facilitated by streamlined governance and the leveraging of informaticians to improve systems. Key points identified include the utility of existing EHR systems in supporting rapid expansion of telehealth, the importance of interoperability and availability of patient data to the provider, and strategies for conserving resources. Although some of these lessons may already have been learned and adopted within digitally mature hospitals, this paper can indicate potential strategies for less digitally mature settings, and for responses to future pandemics of a similar nature. Our interview data point to the value of a combination of responsive governance, EHRs and accompanying technologies to reduce burden of care, decrease discharge time, drug shortages and health worker exposure. As COVID-19 continues to be at the forefront of global healthcare priorities, more health systems can begin to consider how best to utilize HIT in the fight against the disease.

**Conflicts of interest**

Dr. Bates reports grants and personal fees from EarlySense, personal fees from CDI Negev, equity from ValeraHealth, equity from Clew, equity from MDClone, personal fees and equity from AESOP, and grants from IBM Watson Health, outside the submitted work. Dr. Sheikh is a member of the Scottish Government Chief Medical Officer's COVID-19 Advisory Group. The remaining authors have no conflicts of interest to declare.

**Data availability statement**

The data underlying this article will be shared on reasonable request to the corresponding author.

**Acknowledgements**

We thank all the participating sites and the individuals who participated in an interview for this study. We thank Serena Tricarico, Kieran Turner, Toni Wigglesworth, and our Patient and Public

Involvement representatives, Antony Chuter and Jillian Beggs, for their support and feedback throughout the project. We also acknowledge the support of colleagues from the Department of Health and Social Care, the National Health Service and the Medicines and Healthcare products Regulatory Agency (MHRA). Ann Slee—NHS; Jason Cox—DHSC; Richard Cattell—NHS; Helen Causley—DHSC; Paul Stonebrook—DHSC; Mick Foy—MHRA; Kathryn Ord—MHRA; Graeme Kirkpatrick—NHS. We thank the referees for reviewing this manuscript.

**Funding**

This study/project is funded by the National Institute for Health Research (NIHR) [Optimising ePrescribing in Hospitals (PR-ST-01-10001)/Policy Research Programme]. The views expressed are those of the author(s) and not necessarily those of the NIHR or the Department of Health and Social Care.

**Contributorship**

AS conceptualized the wider study and secured the funding. CH and SM conducted data collection and analysis. SM prepared the first draft of the manuscript. SM and CH prepared the second draft of the manuscript. SM, CH, DB and AS drafted the final version of the manuscript. All authors reviewed and approved the final version prior to submission
